# Exploring the clinical utility of two staging models for bipolar disorder

**DOI:** 10.1111/bdi.12825

**Published:** 2019-09-04

**Authors:** Afra van der Markt, Ursula M. H. Klumpers, Annemiek Dols, Stasja Draisma, Marco P. Boks, Annet van Bergen, Roel A. Ophoff, Aartjan T. F. Beekman, Ralph W. Kupka

**Affiliations:** ^1^ Psychiatry Amsterdam UMC Vrije Universiteit Amsterdam Amsterdam Public Health Research Institute Amsterdam The Netherlands; ^2^ Psychiatry, Amsterdam Neuroscience Amsterdam UMC Vrije Universiteit Amsterdam Amsterdam The Netherlands; ^3^ GGZ inGeest Specialized Mental Health Care Amsterdam The Netherlands; ^4^ Department of Psychiatry University Medical Center Utrecht Utrecht The Netherlands; ^5^ Department of Psychiatry Brain Center Rudolf Magnus University Medical Center Utrecht Utrecht The Netherlands; ^6^ Center for Neurobehavioral Genetics Semel Institute for Neuroscience and Human Behavior University of California Los Angeles Los Angeles CA USA; ^7^ Department of Human Genetics University of California Los Angeles CA USA

**Keywords:** bipolar disorder, mood episodes, staging, staging models

## Abstract

**Objective:**

To assess the clinical utility of two staging models for bipolar disorder by examining distribution, correlation, and the relationship to external criteria. These are primarily defined by the recurrence of mood episodes (model A), or by intra‐episodic functioning (model B).

**Methods:**

In the Dutch Bipolar Cohort, stages according to models A and B were assigned to all patients with bipolar‐I‐disorder (BD‐I; N = 1396). The dispersion of subjects over the stages was assessed and the association between the two models calculated. For both models, change in several clinical markers were concordant with the stage was investigated.

**Results:**

Staging was possible in 87% of subjects for model A and 75% for model B. For model A, 1079 participants (93%) were assigned to stage 3c (recurrent episodes). Subdividing stage 3c with cut‐offs at 5 and 10 episodes resulted in subgroups containing 242, 510, and 327 subjects. For model B, most participants were assigned to stage II (intra‐episodic symptoms, N = 431 (41%)) or stage III (inability to work, N = 451 (43%)). A low association between models was found. For both models, the clinical markers “age at onset,” “treatment resistance,” and “episode acceleration” changed concordant with the stages.

**Conclusion:**

The majority of patients with BD‐I clustered in recurrent stage 3 of Model A. Model B showed a larger dispersion. The stepwise change in several clinical markers supports the construct validity of both models. Combining the two staging models and sub‐differentiating the recurrent stage into categories with cut‐offs at 5 and 10 lifetime episodes improves the clinical utility of staging for individual patients.

## 1 INTRODUCTION

Clinical staging models are widely used in medicine to classify disease progression in chronic conditions such as cancer, cardiac failure, autoimmune disorders, and dementia.^1^ Only relatively recently has the concept of staging been introduced in the field of psychiatry, first by Fava and Kellner,^2^ later by McGorry et al.^3^

Bipolar disorder (BD) is characterized by recurrent (hypo)manic and depressive episodes alternating with euthymic intervals and has a highly heterogeneous longitudinal course and outcome. Diagnostic and prognostic precision would therefore benefit from a staging system that determines and predicts illness progression in individual patients that can guide treatment decisions as early as possible. Given the lack of established biomarkers underpinning the pathophysiology, current staging models in psychiatry rely exclusively on clinical characteristics.

In recent years, numerous studies have aimed at identifying biomarkers for bipolar disorder, and some progress has been made. Teixeira et al^4^ reviewed the current state of peripheral (blood‐derived), genetic, neuroimaging, and neurophysiological candidates for biomarkers of bipolar disorders and found several promising candidates, including peripheral inflammation markers and Brain‐Derived Neurotrophic Factor (BDNF). Several studies have found bipolar illness to be associated with chronic low‐grade inflammation with exacerbations during mood episodes.^5^, ^6^, ^7^, ^8^ A decrease in BDNF was found during manic and depressive episodes. High BDNF levels were found to be associated with a good treatment response to lithium.^9^, ^10^ Other interesting candidates include oxidative stress markers such as lipid peroxidation, DNA/RNA damage and nitric oxide, neuronal markers such as S100B and Neuron Specific Enolase (NSE), and metabolic markers such as GLP‐1, ghrelin, adiponectin, and GIP.^11^ No specific genetic maker for BD has been identified, although BD has high heritability.^12^, ^13^ The current consensus implicates a role of multiple genetic variants that are dependent on environmental interactions and epigenetic mechanisms. When combined, they increase the chance of developing BD.^4^ Most neuroimaging studies found alterations in cortical thickness similar to those in schizophrenia and major depressive disorder. Several fMRI studies found a different activity in brain regions responsible for emotional regulation and cognitive control.^4^ Various candidates for neurophysiological biomarkers have been identified, using electroencephalography (EEG) or magnetoencephalography (MEG), such as lower delta inter‐hemispheric coherence in the frontal region and greater parietal‐temporal and central parietal region alpha hemispheric coherence,^14^ suggesting dysfunctional long‐range cortical connectivity in BD.

Two staging models prevail for BD. The model as proposed by Berk et al 15 (further called “model A” in our paper) is largely defined by the occurrence and recurrence of mood episodes. It starts with an at‐risk stage (0), moving from a prodromal stage 1 to first episode stage 2, to recurrent stage 3 to chronic unremitting illness stage 4. The model is fueled by the neuroprogression hypothesis that every mood episode is toxic to the brain.^16^ We previously applied this model to patients in order to evaluate the stage progression over the course of the first 5 years after the diagnosis of BD.^17^ Frank et al^18^ applied this model to subjects with BD to assess genetic and neuroimaging markers for each stage, after which progressive changes were found.

Kapczinski et al^19^ proposed an alternative staging model (‘model B’) based upon intra‐episodic functional impairment. This includes a latent stage and four clinical stages, defined by the absence of intra‐episodic symptoms (stage I), intra‐episodic symptoms (stage II), intra‐episodic impairment with inability to work (stage III), and inability to live autonomously (stage IV). Model B is based on McEwan and Stellar´s^20^ concept of allostatic load, attributing cognitive damage to the attempt of an individual to deal with chronic exposure to fluctuating or heightened neural or neuroendocrine activity. When this model was applied to outpatients, Goi et al^21^, ^22^ found a decrease in treatment response with increasing stages and Rosa et al^23^ found progressive neuropsychological and functional changes from stage I to stage IV, with greater impairment in later stages of the illness.

Both models take a complementary perspective on illness progression, and are studied separately,^17^, ^24^, ^25^ but not in relationship to each other. External criteria have only been tested for each model separately.

A third model was proposed by Duffy et al^26^ This model is not suitable for the aim of this study since it focuses on the early stages of bipolar disorder, whereas our sample consisted of patients with established bipolar I disorder. Post et al^27^ proposed a combined staging model which largely overlaps with both model A and B.

In this study, we have investigated both models next to each other using data from the Dutch Bipolar Cohort. For a staging model to have clinical utility, sufficient distribution over the stages is necessary, since this is a measure for the distinctiveness of the model and the ability to define illness progression. Our first aim was therefore to assess the dispersion of the subjects over the different stages for both models. We hypothesized to find a lower dispersion in Model A over Model B, as Model A will likely exhibit a ceiling effect as all nonchronic subjects with recurrent (ie, two or more) mood episodes are assigned to the same stage.

Second, the association between the two models was determined. Since both models are based on an underlying concept of illness progression, we expected to find a high association between both models. Alternatively, a low association would suggest that both models reflect different aspects of the illness, supporting the idea that a combination of models would be synergetic.

However, both models may represent different indicators of the underlying progress of BD. Given a lack of specific biological markers, the validity of both models may be compared using clinical markers of disease progression, such as age at onset, BD in parents, childhood trauma, treatment resistance, and longitudinal illness course. We expected to find alterations in these markers concordant with the stages in the two models.

## 2 METHOD

### 2.1 Participants and procedure

Data were acquired from the Dutch Bipolar Cohort (DBC), performed by the University Medical Center Utrecht (UMCU), the Netherlands, in collaboration with the University of California at Los Angeles (UCLA). Patients were recruited via clinicians (19.2%), the Dutch BD patient association (15.8%), pharmacies (33.6%), advertisements (6.9%), self‐referral (5%), participated in previous studies of the UMCU (4.5%), or from miscellaneous undocumented resources (15.0%).^28^ The diagnosis bipolar‐I‐disorder was confirmed using the Structured Clinical Interview for DSM‐IV (SCID‐I; 29).

Inclusion criteria for all participants were: a minimum age of 18 years at inclusion, at least three grandparents of Dutch ancestry, and a thorough understanding of the Dutch language. The study was approved by the medical ethical committee of the UMCU and all participants gave written informed consent.

Clinical interviews were conducted and the Questionnaire for Bipolar Disorders (QBP‐NL, Akkerhuis et al^30^) was completed. The QBP‐NL is an adaption and Dutch translation of the Enrolment Questionnaire as previously used in the Stanley Foundation Bipolar Network by Leverich et al^31^ and Suppes et al.^32^

More information on this cohort is provided in the studies of Vreeker et al^33^ and Van Bergen et al.^28^ The sample characteristics are presented in Table 1.

**Table 1:** Sociodemographic and clinical characteristics of participants (N = 1396)

Descriptives	Mean (SD)[range]	N(%)
Age in years	51.1 (13.8) [18.4–91.3]	
Onset of mood symptoms^a^		
depressive symptoms	24.6 (11.1) [1–70]	
manic symptoms	29.0 (11.3) [2–63]	
Sex, m/f		603/793 (43)
Education^a^		
primary school		136 (9.8)
secondary school		611 (44.1)
higher education		639 (46.1)
Previous depressive episodes^a^		
0		29 (2)
1–5		627 (45)
6–10		176 (13)
11–20		108 (8)
>20		46 (3)
Don't know/missing		410 (29)
Previous manic/hypomanic episodes^a^		
0		0 (0)
1–5		955 (68)
6–10		161 (12)
11–20		78 (6)
>20		29 (2)
Don't know/missing		173 (12)
Medication at inclusion		
any		1308 (97)
lithium		1205 (90)
valproic acid		356 (26)
antipsychotics		967 (72)
antidepressants		692 (51)
Childhood abuse^a^		
physical		384 (28)
sexual		322 (23)
both		137 (10)

a self‐reported.

### 2.2 Application procedure for staging

**Figure 1:**
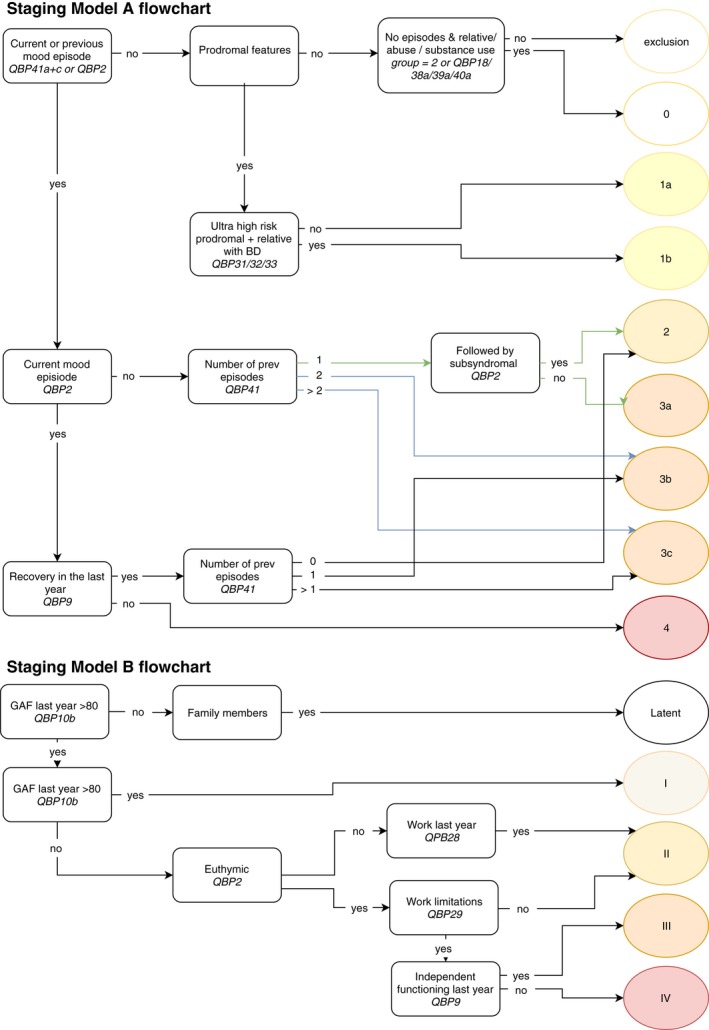
Flowchart of two models [Colour figure can be viewed at http://wileyonlinelibrary.com]

All subjects were assigned to a stage from both model A and model B using a decision flowchart (Figure 1).

For model A, (sub)stages were allocated using a set number of items originating from the Questionnaire for Bipolar Disorder (QBP; 30). Patients were divided into groups on the basis of the current mood episode. Those with a current mood episode were divided into two groups, euthymia in the previous year versus the absence of a period of recovery in the last year, the latter qualifying for stage 4. The total lifetime number of previous manic and depressive episodes was summed. In case of a current mood episode, one additional mood episode was added to the total. Patients were allocated to stage 2 (one mood episode), stage 3a (one mood episode with current residual symptoms), stage 3b (two mood episodes) and stage 3c (multiple recurrent mood episodes). Since the majority of subjects were allocated to group 3c, this stage was further subdivided, with cut‐off points at 5 and 10 episodes, in accordance with cut‐off points previously defined by Berk et al and Magalhães et al.^16^, ^34^

For model B,^19^ subjects were assigned to a stage ranging from latent to stage IV, using a predetermined set of items from the Questionnaire for Bipolar Disorder (QBP; 30) (Figure 1). Stages I to IV were assigned based on social, occupational, and psychological functioning (GAF > or <80), current mood episode (yes or no), employment over the last year (yes or no), work limitations (present or not present), and limitations in functioning (present or not present).

### 2.3 External criteria

Several markers for clinical disease progression were assessed. These were selected based on earlier recommendations^24^ including BD in parents, childhood physical abuse, childhood sexual abuse, age at onset, episode acceleration, increasing or decreasing episode severity and treatment resistance (Table 2). Treatment resistance was operationalized as the currently used number of classes of pharmacotherapeutic interventions (use of classic mood stabilizer, antipsychotics, antidepressants). According to international guidelines for the treatment of bipolar disorder, monotherapy is preferred as compared to polypharmacy, therefore polypharmacy may indicate those patients who are less responsive to medication. The following aspects of the longitudinal illness course were examined: acceleration (ie, less time between episodes) and alterations (increase or decrease) in episode severity over time, scored on a 5‐point Likert scale in the QBP. Childhood physical and sexual abuse were self‐rated on a 4‐point Likert scale in the QBP, ranging from never to often.

### 2.4 Statistics

Data were analyzed using SPSS24.0 35. A Spearman's rank correlation was calculated as a measure of association between models. The markers for clinical disease progression were tested for construct validity using analysis of variance (ANOVAs) and X^2^ statistics.

## 3 RESULTS

### 3.1 Clinical sample

The sample consisted of 1396 patients. All demographic and clinical variables and test statistics are listed in Table 1.

### 3.2 Classification and dispersion

For model A, 1218 of 1396 patients (87%) could be allocated to a stage; for model B, 1050 of 1396 patients (75%) were assigned to a stage. Non‐allocation was mainly due to missing data in the dataset, ie missing answers in the QBP.

As can be expected from a clinical sample, for model A, stage 0 (at risk) and stage 1 (prodromal) were not available; 10 subjects classified as stage 2 (first full episode), 18 as stage 3a, 55 as stage 3b, and 1079 (93%) as stage 3c (recurrent episodes). Subdividing stage 3c into groups with ≤5 lifetime mood episodes, 6–10 episodes, and >10 episodes resulted in subgroups containing 242, 510, and 327 subjects. Stage 4 (chronicity) held 56 subjects. For model B, no subjects were categorized as latent. Subjects 79, 431, 456, and 84, respectively, were in stages I (full recovery), II (intra‐episodic symptoms), III (inability to work), and IV (inability to live autonomously).

After combining the two models, 1002 patients could be assigned to both staging models. The dispersion of subjects over the stages was assessed. The majority of subjects clustered for model A in stage 3c (multiple recurrences; N = 917) and for model B in stages II (inter‐episodic symptoms; N = 396) and III (inability to work; N = 451) (see Table 3). As expected, the most distinctive parameter in model A was the number of episodes. The most distinctive parameter for model B, for stages II and III was the ability to work, but not the current work situation. The ability to work did not differentiate for stage II: 99% stated to have no or only mild work limitations, but only 82% of patients were actually working. Moreover, 1% stated to have severe work limitations, but 18% were not working. For stage III 36% had no or only mild limitations and 64% had severe limitations, but despite this large group with limitations 64% was employed and 36% was not.

### 3.3 Association between models

A Spearman's rank correlation was calculated between model A and B, with stage 3c of model A subdivided in cutoffs at 5 and 10 episodes (as shown in Table 3). The correlation for models A and B was 0.21 (*P* < .05), signifying a low association.

### 3.4 Clinical markers

For both model A and model B, clinical markers age at onset, episode acceleration, and treatment resistance changed significantly over the stages. For model A, both increased and decreased episode severity changed significantly over stages.

**Table 2:** Assessment of change in clinical indicators

	Model A			Model B		
	ANOVA (F)	Chi‐sq (X^2^)	*P*	ANOVA (F)	Chi‐sq (X^2^)	*P*
Age at onset	13.9		<.01*	4.6		<.01*
Bipolar parents		2.3	.68		.2	.97
Childhood trauma						
Childhood physical abuse		14.9	.25		11.8	.27
Childhood sexual abuse		8.1	.77		15.7	.08
Trajectories						
Episode acceleration		23.5	.02*		36.7	<.01*
Increase in episode severity		28.8	.04*		13.9	.13
Decrease in episode severity		29.3	<.01*		4.8	.85
Treatment resistance		39.7	<.01*		22.2	.01*

* significant, *P* < .05.

**Table 3:** Dispersion over two models

Staging Model A (Berk et al, 2007(15))		Staging Model B (Kapczinski et al, 2009(19))					Total
		Latent	Stage I	Stage II	Stage III	Stage IV	
		At risk	Full recovery	Inter‐episodic symptoms	Inter‐episodic Impairment	Inability living autonomously	
Stage 0	Increased risk	0	0	0	0	0	0
Stage 1	Nonspecific	0	0	0	0	0	0
Stage 2	Threshold episode	0	0	4	2	0	6
Stage 3a	Subthreshold recurrence	0	6	3	4	0	13
Stage 3b	Recurrence	0	8	25	12	2	47
Stage 3c	Multiple recurrences	0	56	348	433	80	917
	<5 episodes	0	19	114	70	13	216
	6‐10 episodes	0	24	167	203	34	428
	> 10 episodes	0	13	67	160	33	273
Stage 4	Unremitting illness	0	3	16	0	0	19
Total		0	73	396	451	82	1002

ρ: Spearman's rank correlation 0.21 (*P* < .05).

## 4 DISCUSSION

Using data from the Dutch Bipolar Cohort, we investigated the applicability of two different staging models for subjects with BD type I, to determine their clinical utility. These two staging models approach illness progression from different and complementary perspectives: recurrence of mood episodes (model A), versus inter‐episodic functional decline (model B), supported by our finding of a low association between the models. In our clinical sample, we found a high degree of clustering in a few stages.

Different criteria can be used to measure the clinical utility of a staging model. Berk et al^24^ stated that “The utility and validity of a staging model for BD depends on its linking to clinical outcome, treatment response, and neurobiological measures.” We suggest adding dispersion as a measure of utility, since this reflects the distinctiveness of a model, that is, if subjects cluster within only one or two stages, the models’ utility is limited for that population. Our substantial sample, representative of Dutch outpatients treated for bipolar‐I‐disorder, allowed us to assess the dispersion of subjects over the various stages, except for the presyndromal stages of both models for obvious reasons. We found a vast majority of subjects clustering in model A, stage 3c, defined as more than one recurrent episode after remission during the previous year, due to multiple relapses of mood episodes. For model B, clustering occurred mainly in stages II and III, defined by intra‐episodic mood symptoms, and (in)ability to work (II, yes) or (III, no). Still, a substantial number of subjects were allocated to stage I and IV, indicating model B to be more distinctive than model A in this patient group. This clustering for both models will likely be the case for most BD patients in specialized care. Dispersion will be larger by using a further subdivision of stage 3c, as in cut‐offs at 5 and 10 episodes, as previously shown by Magalhães et al^34^ and Berk et al.^36^ The clinical utility for this subdivision has previously been found to align with episode dependent treatment resistance.^36^, ^37^ After 10 episodes, a decrease in mood‐stabilizing response was found for lithium by Swann et al.^37^ Berk et al 36 found cut‐offs at 5 and 10 episodes for a diminishing response to olanzapine.

Subdividing stage 3c into patient groups with a maximum of 5 episodes, 6 to 10 episodes and more than 10 episodes resulted in larger dispersion, potentially indicating different illness trajectories.

Although we found a significant measure of association between the two models, a correlation of .21 suggests that models A and B are based on different aspects of the illness. Although both models presume an underlying concept of illness progression, model A is based on the dysfunction of mood regulation in the brain, reflected by episode recurrence, and model B is based on allostatic load with consecutive cognitive damage due to increased neural or endocrine activity and inability to fully recover between episodes**.** Since both mechanisms may play a varying role in individual patients, combining both models might lead to a more accurate staging model.

An interesting finding is that a number of subjects (N = 73) experienced recurrent episodes, without an apparent decline in inter‐episodic functioning. This suggests that there are different illness trajectories with more or with less favorable outcomes. It would be interesting to test which factors determine the increased resilience in these individuals, opening opportunities for treatment strategies.

Ideally, for a clinical useful staging model, clinical markers show an alteration concordant with the staging model.^24^ In both models, the markers “age at onset,” “episode acceleration,” and “treatment resistance” show an increase concordant with the stages, supporting the construct validity of the staging model. We found “age at onset” to lower in higher stages in both models, underscoring onset at childhood or adolescence to be associated with worse outcome.^38^, ^39^ We found episode acceleration over progressive stages, indicating less time to recover or remain well. This is in line with the kindling model.^40^ The increasing number of medication classes over the stages is in line with earlier studies on treatment resistance.^21^, ^37^ Future studies may focus on selecting those variables putting individuals at a higher risk of progressing to more advanced stages, adjusting clinical interventions accordingly.

Our study, as well as the staging models as such, may have several limitations. Both models are based on clinical parameters since there are no validated biomarkers for BD. Both models are designed with the intent to be applied to actual patients instead of an existing database, therefore the fit between these models and the use in clinical settings can only be approximated. Both models are unsuitable to capture subtle differences in disease progression since each category still contains a broad range of clinical manifestations. A possible limitation of staging model B is that psychosocial functioning may be rated differently depending on the cultural context. Eg independent functioning will be evaluated differently in a society where it is more common to live with family as compared to living alone, and work demands may largely differ for each country. Another limitation may have been that the approach to assigning stages in model B was not yet validated. Earlier applications of model B by Goi et al^21^, ^22^ and Rosa et al^23^ used a semi‐structural interview and the Functional Assessment Short Test^21^, ^23^ to classify subjects according to model B; we used similar items from the QBP.^31^ A possible restriction of our study was that for model B the level of functioning was based on a limited amount of self‐rated parameters. The generalizability of our outcomes may be limited due to selection bias resulting in a sample of relative high age. The sample may also be less representative for patients with non‐Dutch ancestry since one of the inclusion criteria was to have at least three grandparents of Dutch ancestry. The reported number of previous mood episodes may be subject to recall bias, which may lead to some inaccuracy in assigning the stage for model A. The clinical indicator treatment resistance reflects the number of medication classes per patient, which may be due to a number of reasons, including treatment resistance.

In conclusion, our study supports the concept of illness progression captured in staging models for the majority of BD subjects currently in treatment. The two investigated staging models approach illness progression from a complementary perspective (as shown by the low association between models). We propose to use a unified model which combines both staging models. To assign a stage to an individual patient, the stages from both model A (episode recurrence) and B (interepisode functioning) may be mentioned, and in addition the lifetime number of mood episodes (eg, a patient with Stage 3 from model A and Stage II from model B, and a total of five mood episodes could be written down as A3BII‐5). This could be further specified by dividing model A, stage 3, into groups with cutoffs at 5 and 10 episodes and by adding clinically important markers to the unified staging model. Additional research is needed to further identify the clinical and biological markers that increase the risk of progression to a subsequent stage.
